# METTL3‐mediated N6‐methyladenosine exacerbates ferroptosis via m6A‐IGF2BP2‐dependent mitochondrial metabolic reprogramming in sepsis‐induced acute lung injury

**DOI:** 10.1002/ctm2.1389

**Published:** 2023-09-15

**Authors:** Hao Zhang, Dan Wu, Yanghanzhao Wang, Kefang Guo, Charles B. Spencer, Lilibeth Ortoga, Mengdi Qu, Yuxin Shi, Yuwen Shao, Zhiping Wang, Juan P. Cata, Changhong Miao

**Affiliations:** ^1^ Department of Anesthesiology Zhongshan Hospital Fudan University Shanghai China; ^2^ Shanghai Key Laboratory of Perioperative Stress and Protection Shanghai China; ^3^ Department of Anesthesiology Shanghai Medical College Fudan University Shanghai China; ^4^ Department of Cardiac Surgery Ohio State University Columbus Ohio USA; ^5^ Department of Biomedical Engineering Ohio State University Columbus Ohio USA; ^6^ Department of Anesthesiology Affiliated Hospital of Xuzhou Medical University Xuzhou China; ^7^ Department of Anesthesiology and Perioperative Medicine University of Texas‐MD Anderson Cancer Center Houston Texas USA; ^8^ Anesthesiology and Surgical Oncology Research Group Houston Texas USA

**Keywords:** ferroptosis, metabolic reprogramming, N6‐methylation, neutrophil extracellular traps, sepsis‐induced acute lung injury

## Abstract

Neutrophil extracellular traps (NETs), released by polymorphonuclear neutrophils (PMNs), exert a robust antimicrobial function in infectious diseases such as sepsis. NETs also contribute to the pathogenesis and exacerbation of sepsis. Although the lung is highly vulnerable to infections, few studies have explored the role of NETs in sepsis‐induced acute lung injury (SI‐ALI). We demonstrate that NETs induce SI‐ALI via enhanced ferroptosis in alveolar epithelial cells. Our findings reveal that the excessive release of NETs in patients and mice with SI‐ALI is accompanied by upregulation of ferroptosis depending on METTL3‐induced m6A modification of hypoxia‐inducible factor‐1α (HIF‐1α) and subsequent mitochondrial metabolic reprogramming. In addition to conducting METTL3 overexpression and knockdown experiments in vitro, we also investigated the impact of ferroptosis on SI‐ALI caused by NETs in a caecum ligation and puncture (CLP)‐induced SI‐ALI model using METTL3 condition knockout (CKO) mice and wild‐type mice. Our results indicate the crucial role of NETs in the progression of SI‐ALI via NET‐activated METTL3 m6A‐IGF2BP2‐dependent m6A modification of HIF‐1α, which further contributes to metabolic reprogramming and ferroptosis in alveolar epithelial cells.

## Graphical abstract

**Highlights**

- NETs induce ferroptosis in alveolar epithelial cells via activation of METTL3‐mediated m6A modification.
- METTL3 induces HIF‐1α upregulation via m6A‐IGF2BP2‐dependent mechanism to exacerbate ferroptosis.
- METTL3 induces enhanced glycolysis and decreased oxidative phosphorylation in alveolar epithelial cells.
- Inhibiting NET formation via peptidyl arginine deiminase 4 knockout attenuates ferroptosis and sepsis‐associated lung damage in mice.

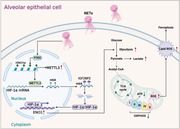

## 1 INTRODUCTION

Acute lung injury (ALI) and acute respiratory distress syndrome (ARDS) are life‐threatening disorders associated with high morbidity and mortality rates.^1^ Numerous interventions to treat ALI/ARDS, such as administering dexamethasone intravenously and low tidal volume ventilation, have not improved survival.^2^, ^3^, ^4^ Hence, the mortality associated with ALI/ARDS remains between 30% and 40%, representing 10% of all deaths globally in intensive care units (ICUs).^5^ In the wake of the COVID‐19 pandemic, the incidence and severity of ALI/ARDS have further increased, and effective therapeutic strategies are urgently required.^6^ In addition, patients who survive ALI/ARDS face a poor prognosis and a long period of poor quality of life.^7^

It has been increasingly acknowledged that sepsis is one of the leading causes of ALI/ARDS.^8^ An uncontrolled inflammatory response and endothelial barrier dysfunction characterise sepsis and ALI/ARDS.^9^, ^10^ During the progression of the disease, polymorphonuclear neutrophils (PMNs) are the first line of immune defence against pathogens.^11^ In addition to their well‐known defence mechanisms, such as phagocytosis and degranulation, neutrophils can release neutrophil extracellular traps (NETs)—webs of DNA material decorated with bactericidal proteins—to efficiently ensnare microorganisms in the circulation.^12^ However, excessive NET components can also serve as damage‐associated molecular patterns (DAMPs) that induce prolonged inflammation and endothelial damage.^13^ NETs also participate in the intravascular coagulation caused by sepsis.^14^ Specifically, an activated NET–platelet–thrombin axis triggers clotting, further contributing to microvascular dysfunction and ischaemic injury of end organs.^15^ Notably, NET‐enhanced glycolysis exacerbates sepsis‐induced ALI (SI‐ALI).^16^, ^17^

Ferroptosis is an iron‐dependent form of regulated cell death, featured by redox imbalance and subsequent toxic lipid peroxidation.^18^ Additionally, several studies have demonstrated the crucial role of mitochondria in ferroptosis, suggesting metabolic alterations during this process.^19^, ^20^ In recent years, it has been gradually recognised that ferroptosis correlates with the occurrence and progression of various diseases, including ALI.^21^ m6A modification has recently attracted attention because of its vital role in gene expression regulation.^22^, ^23^, ^24^, ^25^ In our previous study, we identified that m6A levels are significantly upregulated in NET‐treated alveolar epithelial cells in vitro and by modulating METTL3 expression through knockdown or overexpression in alveolar epithelial cells, we demonstrated that METTL3 is a crucial contributor to m6A levels.^16^, ^26^ Intriguingly, NETs and ferroptosis are tightly connected to metabolic reprogramming, positively correlating to disease severity. These results indicate that NET‐induced m6A modification promotes ferroptosis during ALI, which depends on metabolic changes. However, the underlying molecular mechanism is unclear.

The present study provides evidence that NETs strongly correlate with disease severity in SI‐ALI patients. Moreover, we further uncovered a novel mechanism of ferroptosis via m6A‐IGF2BP2‐dependent mitochondrial metabolic reprogramming. We also evaluated the therapeutic potential of targeting NETs in the context of SI‐ALI.

## 2 MATERIALS AND METHODS

### 2.1 Ethnic statements and patients

Blood samples were obtained from healthy donors and septic patients according to the protocol approved by the Ethics Committee of Zhongshan Hospital, Fudan University (B2021‐182R). Written informed consent was obtained from all participants or their relatives. In detail, 10 mL of peripheral venous blood was collected from septic patients within 1 h of ICU admission. Patients’ treatment outcomes were recorded after hospitalised in the ICU. ‘Dead’ patients were defined as final non‐survive outcomes hospitalised in the ICU. ‘Alive’ patients were defined as final survival outcomes hospitalised in the ICU. Mouse experiments were performed following institutional protocols approved by the animal review committee of Zhongshan Hospital, Fudan University (Protocol license number: 2020‐119).

### 2.2 Experimental animals and validation

In detail, METTL3 floxed (METTL3flox/flox) mice were generated via the CRISPR–Cas9 system at the Shanghai Model Organisms Center, Inc. METTL3flox/flox mice were crossed with the tamoxifen‐inducible Sftpc‐CreERT2 mice provided by the Shanghai Model Organisms Center. To generate type II alveolar epithelial cells in METTL3‐deficient mice, the Sftpc‐CreERT2 mice were crossed with METTL3flox/flox mice to obtain Sftpc‐CreERT2(+)‐METTL3flox/flox (METTL3 CKO) mice. Sftpc‐CreERT2(−)‐METTL3flox/flox (METTL3‐C) mice from littermates were used as controls. Male mice (8–10 weeks old) were treated with tamoxifen (10 mg/kg) dissolved in corn oil intraperitoneally for five consecutive days before experiments. Primary type II alveolar epithelial cells were isolated and purified from mice by membrane filtration and immune adhesion according to the previous description for METTL3 knockout (KO) validation.^27–30^ More information strategies for METTL3 CKO mice validation are listed in Figure S4. Peptidyl arginine deiminase 4 (PAD4) KO mice used in the experiments were purchased from Shanghai Model Organisms Co. Ltd. All mice were on a C57BL/6J background and housed in a specific pathogen‐free facility under 12 h light/dark cycle at a temperature of 24 ± 2°C and humidity between 30% and 70%, with access to food and water ad libitum.

### 2.3 Caecal ligation and puncture model

Eight‐ to 10‐week‐old wild‐type (WT) and METTL3 CKO or PAD4 KO mice were used for experiments. The caecal ligation and puncture operation was performed as previously described.^16^ Briefly, mice were anaesthetised with pentobarbital sodium, and their caecum was separated after exposing the abdominal cavity. Then, the caecum was ligated with a 5‐0 suture, punctured with a 20‐gauge needle, and placed back into the abdominal cavity after squeezing faecal content. Both muscles and skin were sequentially sewn, and .9% saline was given for rehydration. Mice in the sham group received the same surgery except for caecal ligation and puncture. For different groups, the following drugs were injected intraperitoneally: CI‐amidine (50 mg/kg, Sigma–Aldrich), anti‐Ly6G antibody (500 μg/mouse, ab238132, Abcam) and DNase I (5 mg/kg, Sigma–Aldrich). All the mice were euthanised 24 h after surgery.

### 2.4 Isolation of neutrophils and NET production

Blood samples from humans or mice were layered for neutrophil separation and centrifuged at 800 *g* for 30 min at room temperature. The lower band containing neutrophils was carefully aspirated and washed with phosphate buffered saline (PBS), and erythrocytes were removed using an erythrocyte lysing reagent. The remaining cells were resuspended in Dulbecco's modified eagle medium (DMEM, Gibco) containing 10% foetal bovine serum (FBS; Gibco). Then, the isolated neutrophils were seeded in six‐well plates (2 × 10^6^ cells per well) and stimulated with 50 nM phorbol 12‐myristate 13‐acetate (PMA, MKBio) for 4 h. After removing the supernatant, NETs adhered to the bottom were washed down by pipetting 2 mL of DMEM and centrifuged at 1000 *g* for 10 min to remove cell debris.

### 2.5 Quantification of dsDNA, myeloperoxidase–DNA complexes, inflammatory indicators and ferroptosis markers

The marker concentrations were detected using relevant kits according to the manufacturer's instructions. The kits used are listed in Table S1.

### 2.6 Immunofluorescence

Cells were fixed with 4% formaldehyde (Servicebio), blocked with 1% bovine albumin (BSA, Biosharp) and incubated with primary antibodies against citrulline histone H3 (CitH3) (1:100, ab5103, Abcam), myeloperoxidase (MPO) (1:50, AF3667, R&D Systems), VE‐cadherin (1:100, 2500, Cell Signaling Technology), glutathione‐peroxidase 4 (GPX4) (1:200, ab125066, Abcam), hypoxia‐inducible factor‐1α (HIF‐1α) (1:100, 36169, Cell Signaling Technology) and METTL3 (1:100, ab195352, Abcam) at 4°C overnight and F‐actin (1:200, C2201S, Beyotime) at 37°C for 2 h. Paraffin‐embedded lung tissue sections were deparaffinised and rehydrated, blocked in 1% BSA and incubated with primary antibodies against CitH3 and MPO at 4°C overnight. The next day, cells or slides were stained with fluorescent secondary antibodies at room temperature for 1 h. The nuclei were stained with 4',6‐diamidino‐2‐phenylindole (DAPI), and the slices were viewed under a microscope (Olympus).

### 2.7 Scanning/transmission electron microscopy

Neutrophils or lung tissue samples (1 mm × 1 mm × 1 mm) were fixed in 2.5% glutaraldehyde and postfixed with 1% osmic acid for 2 h. Gradient dehydration was carried out using 30%, 50%, 70%, 80%, 90%, and 100% ethanol. Neutrophils were dried and sputter‐coated with gold, and images were taken with a scanning electron microscope (SU8100, Hitachi). The lung tissue samples were embedded in 812 resin and stained with 2% uranyl acetate, and ultrastructural images of mitochondria were obtained using a transmission electron microscope (HT7700, Hitachi).

### 2.8 Histopathological analysis

Paraffin‐embedded tissue sections were deparaffinised in xylene, hydrated with graded ethanol and stained with haematoxylin and eosin. The severity of lung injury was evaluated using a semiquantitative histology scoring system described previously,^16^ which graded four items: haemorrhage, alveolar oedema, thickening of the alveolar septa and leukocyte infiltration. From 0 to 3 (0: normal; 1: mild; 2: moderate; 3: severe), the scores of individual items were summed to obtain the lung injury score.^16^

### 2.9 Immunohistochemistry

Paraffin‐embedded tissue sections were deparaffinised in xylene and hydrated with graded ethanol. After being treated with 3% hydrogen peroxide, the sections were blocked with 5% BSA and incubated with primary antibodies against HIF‐1α (1:50, ab114977, Abcam), METTL3 (1:50, ab195352, Abcam) and GPX4 (1:100, ab125066, Abcam) at 4°C overnight. Then, a secondary antibody conjugated with horseradish peroxidase was used, followed by diaminobenzidine staining and counterstaining with haematoxylin. The slides were analysed under light microscopy (Carl Zeiss).

### 2.10 Masson's trichrome staining

Paraffin‐embedded tissue sections were deparaffinised, hydrated and stained with a Masson dye solution set (Servicebio). Next, slices were treated with xylene, sealed with neutral resin and observed under light microscopy (Carl Zeiss).

### 2.11 Lung wet‐to‐dry ratio

Right lungs were harvested and weighed (wet weight) and dried at 60°C for 48 h (dry weight). The wet weight/dry weight ratio was calculated by dividing the wet weight by the dry weight.

### 2.12 Cell culture and treatments

Human alveolar epithelial cells (HPAEpiC) were obtained from ScienCell Research Laboratories (Carlsbad) and cultured in DMEM/F12 (HyClone) supplemented with 10% FBS at 37°C in a 5% CO_2_ humidified incubator. HPAEpiC cells were transfected with small interfering RNA (siRNA) using Lipofectamine 3000 (Invitrogen). The primer sequences of siRNA are listed in Table S2.

### 2.13 Cell viability assay

Cell viability was assayed using Cell Counting Kit‐8 (Dojindo Corp.) according to the manufacturer's instructions. The results were measured at the wavelength of 450 nm.

### 2.14 Real‐time quantitative PCR

We extracted total RNA from lung tissues or HPAEpiC cells using TRIzol reagent (Invitrogen) and reverse‐transcribed it into cDNA using a PrimeScript RT reagent kit (Takara). Real‐time quantitative PCR (RT‐qPCR) was performed with a TB Green PCR kit (Takara), and we analysed relative mRNA levels with β‐actin as the reference gene. The primer sequences are listed in Table S3.

### 2.15 Western blot

Total proteins were extracted using RIPA lysis buffer (Solarbio) and separated by sodium dodecyl sulphate–polyacrylamide gel electrophoresis and subsequently transferred onto polyvinylidene fluoride membranes. The membranes were blocked with 5% milk and then incubated with primary and secondary antibodies. Signals were detected using an ECL chemiluminescence kit (Tanon). The following primary antibodies were used: anti‐P300 antibody (1:1000, ab14984, Abcam), anti‐METTL3 antibody (1:1000, ab195352, Abcam), anti‐Histone H3 antibody (1:1000, 17168‐1‐AP, Proteintech), anti‐IGF2BP2 (1:1000, ab124930, Abcam), anti‐H3K27ac antibody (1:1000, ab4729, Abcam), anti‐HIF‐1α antibody (1:1000, 36169, Cell Signaling Technology), anti‐GPX4 antibody (1:1000, ab125066, Abcam), anti‐hexokinase 2 (HK2) antibody (1:1000, 22019‐1‐AP, Proteintech), anti‐phosphofructokinase (PFKP) antibody (1:1000, AF7733, Beyotime), anti‐GAPDH antibody (1:2000, 5174, Cell Signaling Technology), anti‐enolase 1 (ENO1) antibody (1:3000, 11204‐1‐AP, Proteintech), anti‐pyruvate kinase M2 (PKM2) antibody (1:1000, 4053, Cell Signaling Technology), anti‐lactate dehydrogenase (LDHA) antibody (1:1000, ab52488, Abcam), anti‐Complex I∼V antibody (1:250, ab110413, Abcam) and anti‐β‐actin antibody (1:3000, 3700, Cell Signaling Technology).

### 2.16 m6A dot blot

Total RNA was extracted using TRIzol reagent (Invitrogen) and purified using Dynabeads mRNA Purification Kit (Invitrogen). The purified mRNA was denatured and dotted on an Amersham Hybond‐N+ membrane and then crosslinked under ultraviolet light for 10 min. Next, the membrane was blocked with 5% BSA and then incubated with the anti‐m6A antibody (1:1000, 202003, Synaptic Systems) and secondary antibody. The m6A levels were detected using an ECL chemiluminescence kit (Tanon).

### 2.17 Chromatin immunoprecipitation

According to the manufacturer's instructions, chromatin immunoprecipitation (ChIP) assays were conducted using a Simple ChIP Plus Sonication Chromatin IP Kit (Cell Signaling Technology). HPAEpiC cells were fixed with 1% formaldehyde at 37°C for 10 min, incubated in a lysis buffer and sonicated to shear genomic DNA. Soluble chromatin was incubated with an anti‐H3K27ac antibody (ab4729, Abcam) and normal rabbit immunoglobulin G (2729, Cell Signaling Technology) at 4°C for 12 h. The immunoprecipitate was bound to protein G magnetic beads, and DNA–protein crosslinking was terminated by incubation at 65°C for 2 h. Finally, the immunoprecipitated DNA sequences were quantified by PCR (Table S4).

### 2.18 METTL3 catalytic site mutant

Plasmids pCDNA3‐FLAG and pCDNA3‐FLAG‐METTL3 were purchased from Addgene. The catalytically dead mutant of METTL3 was conducted from pCDNA3‐FLAG‐METTL3 using QuikChange II Site‐Directed Mutagenesis Kit. The residues 395−398, DPPW, were mutated to APPA.

### 2.19 Plasmid construction

In short, constructing a plasmid involves selecting a suitable restriction endonuclease to digest the vector and then purifying the linearised vector through agarose gel electrophoresis. Amplification of the target fragment using designed primers through PCR, and subsequent purification of the correctly sized fragment using agarose gel electrophoresis. Ligation of the linearised vector and the target fragment using homologous recombination or T4 ligation methods. Transformation and cultivation of the resulting product for 12−16 h. Single colony picking to validate the clone and a selection of positive clones with correct validation for sequencing. Plasmid extraction was performed on the correctly sequenced clone samples to ensure high purity and the absence of endotoxins.

### 2.20 Lentiviral transduction

Plasmids containing transgenes and packaging plasmids were cotransfected into HPAEpic cells using Lipofectamine 3000. Viruses were collected and concentrated after 48 h.^31^, ^32^, ^33^ When HPAEpic cells reached 50%−60% confluence, we infected the cells with concentrated virus and then selected them by antibiotic treatment. For the tetracycline‐inducible (Tet‐on) lentiviral expression, the METTL3 gene was cloned into the inducible Tet‐on lentiviral vector (Beijing Syngentech Co., Ltd.). Tet‐on lentivirus‐infected HPAEpic cells were treated with or without a low (1 μg/mL) or a high dosage of tetracycline (2 μg/mL) (Sigma–Aldrich).

### 2.21 RNA sequencing

Total RNA was obtained from HPAEpiC cells for RNA sequencing (RNA‐seq) analysis. Cells were overexpressing or knocking down METTL3 or transfected with vector. RNA quality and quantity were measured using NanoDropTM ND‐1000, and its integrity was determined using agarose gel electrophoresis. The NEBNextR Poly(a) mRNA magnetic isolation module was used to extract mRNA. RNA libraries were created with KAPA Stranded RNA‐Seq library Preparation kit (Illumina) and sequenced using the Illumina HiSeq 4000 platform.

### 2.22 MeRIP‐qPCR assay and MeRIP‐seq analysis

For methylated RNA Immunoprecipitation sequencing (MeRIP‐seq), the amount of purified total RNA was greater than 120 μg, and the integrity and quantity of each RNA sample were assessed using agarose gel electrophoresis and a NanoDropTM instrument. Intact mRNA was first isolated from total RNA samples using an Arraystar Seq‐StarTM poly(A) mRNA Isolation Kit according to the manufacturer's protocol. The isolated mRNA was chemically fragmented into 100‐nucleotide‐long fragments by incubation in fragmentation buffer (10 mM Zn^2+^ and 10 mM Tris–HCl, pH 7.0), and the size of the fragmented mRNA was confirmed using agarose gel electrophoresis. Then, m6A‐methylated mRNAs were immunoprecipitated with an anti‐N6‐methyladenosine (m6A) antibody (an aliquot of the fragmented mRNAs was kept as input). The major procedures included immunoprecipitation, washing and elution. The eluted m6A mRNA fragments were then concentrated for RNA‐seq library construction. RNA‐seq libraries for the m6A antibody‐enriched mRNAs and input mRNAs were prepared using a KAPA Stranded mRNA‐seq Kit (Illumina). The prepared libraries were diluted to a final concentration of 8 pM, and clusters were generated on an Illumina cBot using a HiSeq 3000/4000 PE Cluster Kit (#PE‐410‐1001, Illumina), followed by sequencing on the Illumina HiSeq 4000. For MeRIP‐seq data analysis, the raw reads were trimmed by Trimmomatic software and aligned to the Ensembl reference genome by HISAT2 software (v2.1.0). The differentially m6A‐RIP‐enriched regions (peaks) between METTL3 and the corresponding control group were analysed by exomePeak software. These differential peaks were annotated using the latest Ensembl database. Sequence motifs are one of the basic functional units of molecular evolution. The algorithms of Multiple EM for Motif Elicitation (MEME) and Discriminative Regular Expression Motif Elicitation (DREME) were used to find motifs among the m6A peak sequences.

### 2.23 Measurement of mitochondrial oxidation and extracellular acidification rate

The extracellular acidification rate (ECAR) and the oxygen consumption rate (OCR) were measured using an XF96 Extracellular Flux Analyser (Seahorse Bioscience). HPAEpiC cells (2 × 10^4^ cells per well) were seeded in an XF96 plate and treated successively with glucose, oligomycin, 2‐Deoxy‐D‐glucose (2‐DG) or oligomycin, Synonyms: Carbonyl cyanide 4‐(trifluoromethoxy)phenylhydrazone (FCCP) and rotenone/antimycin A for measurements of ECAR and OCR, respectively. Seahorse Wave software was used to analyse all the data. ECAR in mpH/min and OCR in pmol/min were recorded.

### 2.24 Statistical analysis

Statistical analysis was conducted using GraphPad Prism 9.0 software (GraphPad). The data are presented as the mean ± standard error of the mean. Two‐tailed Student's *t*‐test or two‐way analysis of variance (ANOVA) followed by Tukey's correction was performed for comparisons. *p* < .05 was considered significant.

## 3 RESULTS

### 3.1 NET level shows a positive correlation with disease severity in sepsis patients

**FIGURE 1:**
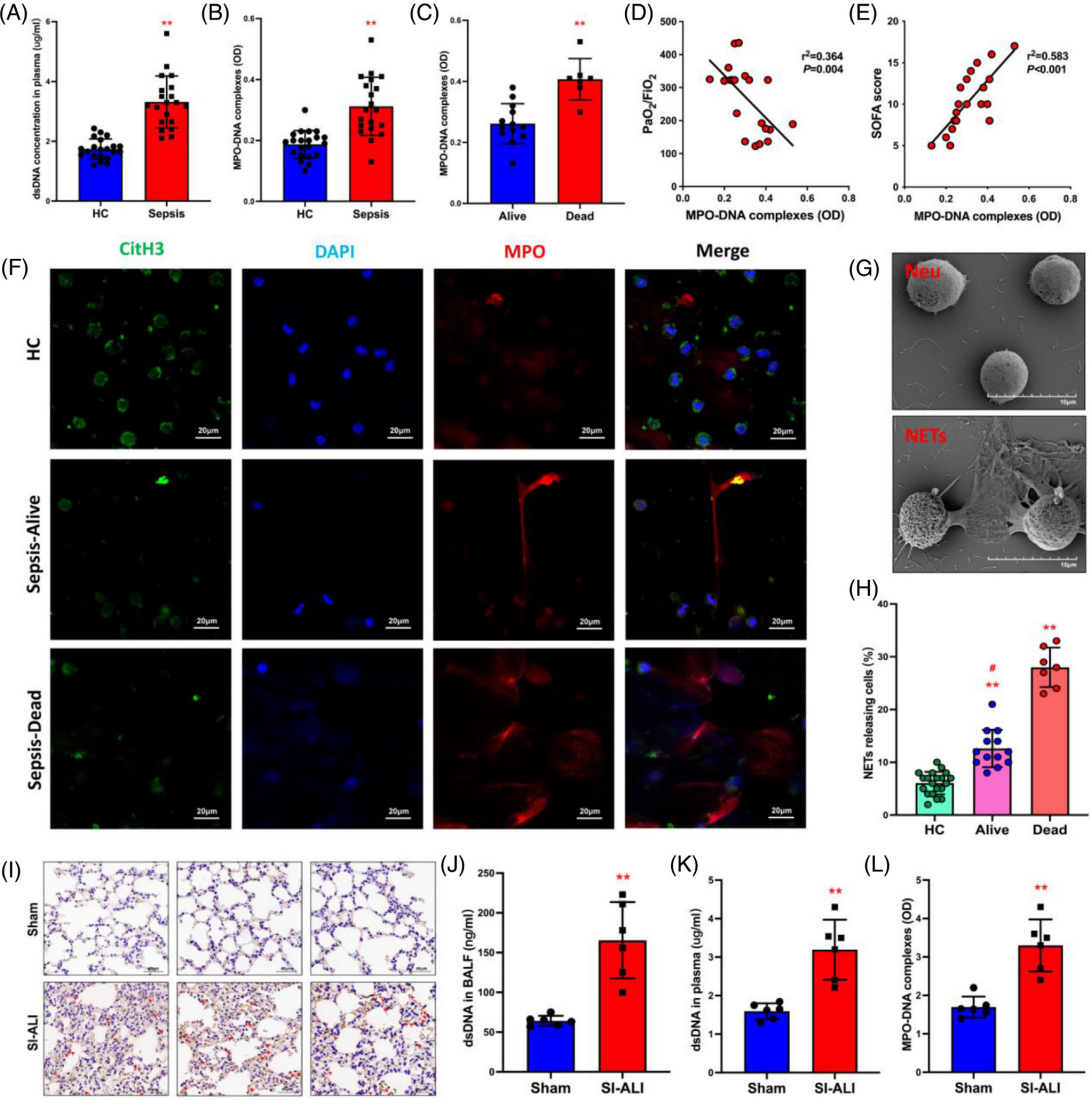
A higher level of neutrophil extracellular traps (NETs) is associated with more severe lung damage and poorer prognosis in patient samples and the acute lung injury (ALI) mouse model. (A) Serum dsDNA and (B) myeloperoxidase (MPO)–DNA complexes were detected in healthy people and patients with sepsis (*n* = 20) and controls (*n* = 20). (C) Serum MPO–DNA complexes were detected in alive and dead sepsis patients. Alive (*n* = 13) and dead (*n* = 7). (D and E) Evaluate the correlation between MPO–DNA complexes and the degree of lung damage (PaO_2_/FiO_2_, SOFA score) in sepsis patients. (F) NETosis representative pictures of alive and dead patients in the controls (red: MPO, green: citrulline histone H3 [CitH3], blue: DAPI; scale bar = 20 μm). (G) Scanning electron microscopy (SEM) images of inactivated and activated neutrophils releasing NETs (scale bar = 10 μm). (H) The percentage of cells releasing NETs from sepsis–dead, sepsis–alive and healthy control (HC) patients. HC (*n* = 20), alive (*n* = 13) and dead (*n* = 7). (I) Representative images of immunohistochemical staining for Ly6G in lung tissues (red arrows; scale bar = 40 μm). (J) dsDNA in bronchoalveolar lavage fluid (BALF), (K) serum dsDNA and (L) serum MPO–DNA complexes were detected in sham and sepsis‐induced ALI (SI‐ALI) mice. Sham (*n* = 6), SI‐ALI (*n* = 6). ^*^ *p* < .05; ^**^ *p* < .01. ‘Dead’ patients refer to those who have final non‐survival outcomes in intensive care unit (ICU). ‘Alive’ patients refer to those who recover and survive in ICU. Two‐way analysis of variance (ANOVA) with Tukey's correction.

Consistent with our previous studies, patients with sepsis showed increased dsDNA (Figure 1A) and MPO–DNA complex (Figure 1B) levels compared with healthy controls. Within patients with sepsis, a higher level of MPO–DNA complex was detected in non‐survivors than in survivors (Figure 1C). Moreover, the amount of MPO–DNA complex was negatively correlated with PaO_2_/FiO_2_ (Figure 1D) and positively associated with sequential organ failure assessment (SOFA) score (Figure 1E). This suggests that high levels of NETs are seen in patients with severe disease. Immunofluorescence (IF) imaging further confirmed that more MPO and CitH3—biomarkers of NET formation—were present in the peripheral blood of not surviving patients (Figure 1F), corresponding with the severity degree of lung damage observed in computed tomography images (Figure S1A). Since NETs were excessively released from neutrophils during persistent or severe infection (Figure 1G), we found that the percentage of cells releasing NETs from non‐surviving patients was increased approximately twofold compared with that in the alive patients and threefold in comparison to that in healthy controls (Figure 1H).

### 3.2 NETs are increased in mice with SI‐ALI

Multiple organ dysfunction, especially ALI, is a hallmark of sepsis. Since patients with high levels of NETs showed more severe lung damage and worse prognosis (Figures 1F–H and S1A), we speculated that neutrophils and NETs played a crucial role in the progression of ALI. To investigate this hypothesis, we turned our attention to a mouse model of sepsis. In the CLP model, our results showed that mice with SI‐ALI had lower survival and higher lung injury scores than the sham group (Figure S1B–D). The SI‐ALI mice displayed increased lung damage, as observed through the destruction of the alveolar wall and significant infiltration of neutrophils (Figure 1I). However, no lymphatic cells were significantly affected (as indicated in Figure S1E–G). As expected, the SI‐ALI group had noticeably elevated levels of dsDNA (Figure 1J–K), and Figure 1L shows that they also had higher MPO–DNA complex levels than the sham group. These results suggest that NETs play a significant role in the development of ALI. Overall, patient and mouse data strongly indicate a direct link between higher levels of NETs and increased disease severity in SI‐ALI.

### 3.3 The inhibition of NETs attenuates lung inflammation in mice with SI‐ALI

Excessive release of NETs may exacerbate tissue damage and cause a persistent inflammatory response. To verify whether NETs could aggravate lung injury and inflammation, we administered inhibitors of NET formation or inducers of NET degradation. Specifically, Cl‐amidine was used to inhibit PAD4—an essential enzyme in NET formation. DNase I was administered to degrade the DNA structures, and the anti‐Ly6G antibody was used to cause neutrophil depletion directly. These treatments diminished NET formation and measurements of dsDNA (Figure S2A–C). The concentration of inflammatory cytokines (tumour necrosis factor [TNF]‐α, interleukin [IL]‐1β and IL‐6) in plasma (Figure S3A–C) and bronchoalveolar lavage fluid (BALF) (Figure S3D–F) also decreased after NET inhibition. These findings demonstrate that inhibiting NET formation protected against lung damage and inflammation in mice with SI‐ALI, further corroborating the importance of NETs during ALI progression.

### 3.4 NETs exacerbate SI‐ALI via induction of ferroptosis in alveolar epithelial cells

**FIGURE 2:**
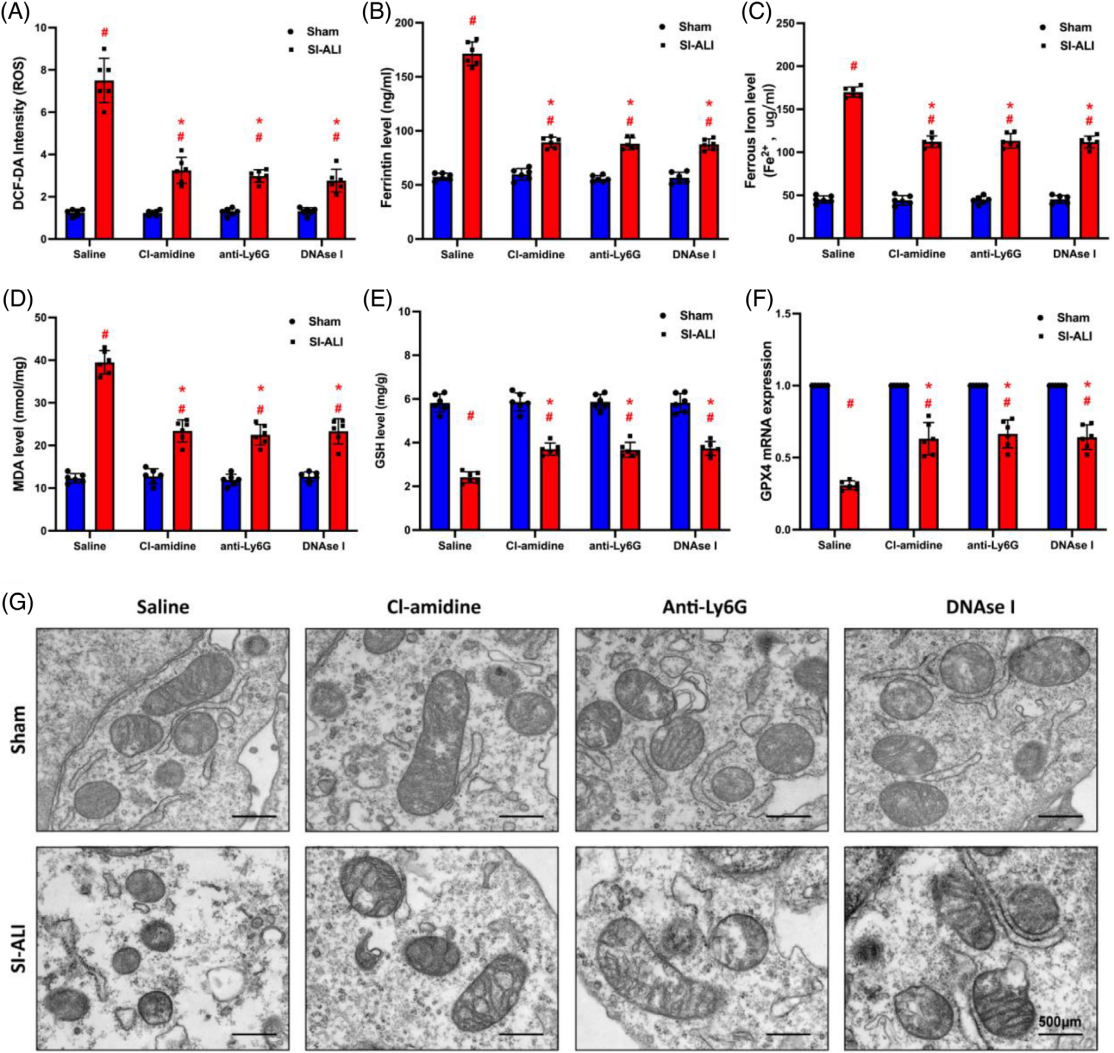
Inhibiting neutrophil extracellular traps (NETs) alleviates ferroptosis in mice with sepsis‐induced acute lung injury (SI‐ALI). We used a CLP‐induced SI‐ALI mouse model. Animals were treated with saline, Cl‐amidine (inhibit NET formation), anti‐Ly6G (deplete neutrophils) and DNase I (degrade NET structures). (A) 2',7'‐Dichlorodihydrofluorescein diacetate (DCF‐DA) assay was used to analyse reactive oxygen species (ROS) levels in mouse lung tissues. (B) ELISA measured the level of ferritin in mouse lung tissues. (C) An iron assay kit detected ferrous iron (Fe^2+^) levels. (D) A lipid peroxidation assay kit measured the level of MDA. (E) ELISA measured the level of glutathione (GSH). (F) Real‐time quantitative PCR (RT‐qPCR) analysed glutathione‐peroxidase 4 (GPX4) mRNA expression level. (G) Images of transmission electron microscopy showed morphological changes in mitochondria (scale bar = 500 μm). ^*^ *p* < .05; ^**^ *p* < .01. ^#^SI‐ALI group versus sham group (two‐way analysis of variance [ANOVA] with Tukey's correction).

Previous research has indicated that ferroptosis is a crucial factor in the development of lung damage caused by sepsis.^16^ In our current model, the levels of reactive oxygen species (ROS) (Figure 2A), ferritin (Figure 2B) and ferrous iron (Figure 2C) increased in SI‐ALI mice. At the same time, they were remarkably suppressed by treatment with Cl‐amidine, anti‐Ly6G antibody or DNase I. Malondialdehyde (MDA), a byproduct of lipid peroxidation in the membrane, exhibited an increase following sepsis. Nonetheless, its level was partially reduced by treatment with Cl‐amidine, anti‐Ly6G antibody or DNase I (Figure 2D). Similarly, glutathione (GSH) levels and GPX4 expression (Figure 2E,F) were reduced in SI‐ALI mice and partially restored by inhibiting NETs. In addition, as shown in Figure 2G, relative to sham group mice, the alveolar epithelial cells of SI‐ALI mice exhibited characteristics typical of ferroptosis; namely, transmission electron microscopy revealed the presence of mitochondrial shrinkage, reduced mitochondrial size and smaller cristae. Mitochondrial damage was found to be relieved with the inhibition of NETs.^19^, ^34^

**FIGURE 3:**
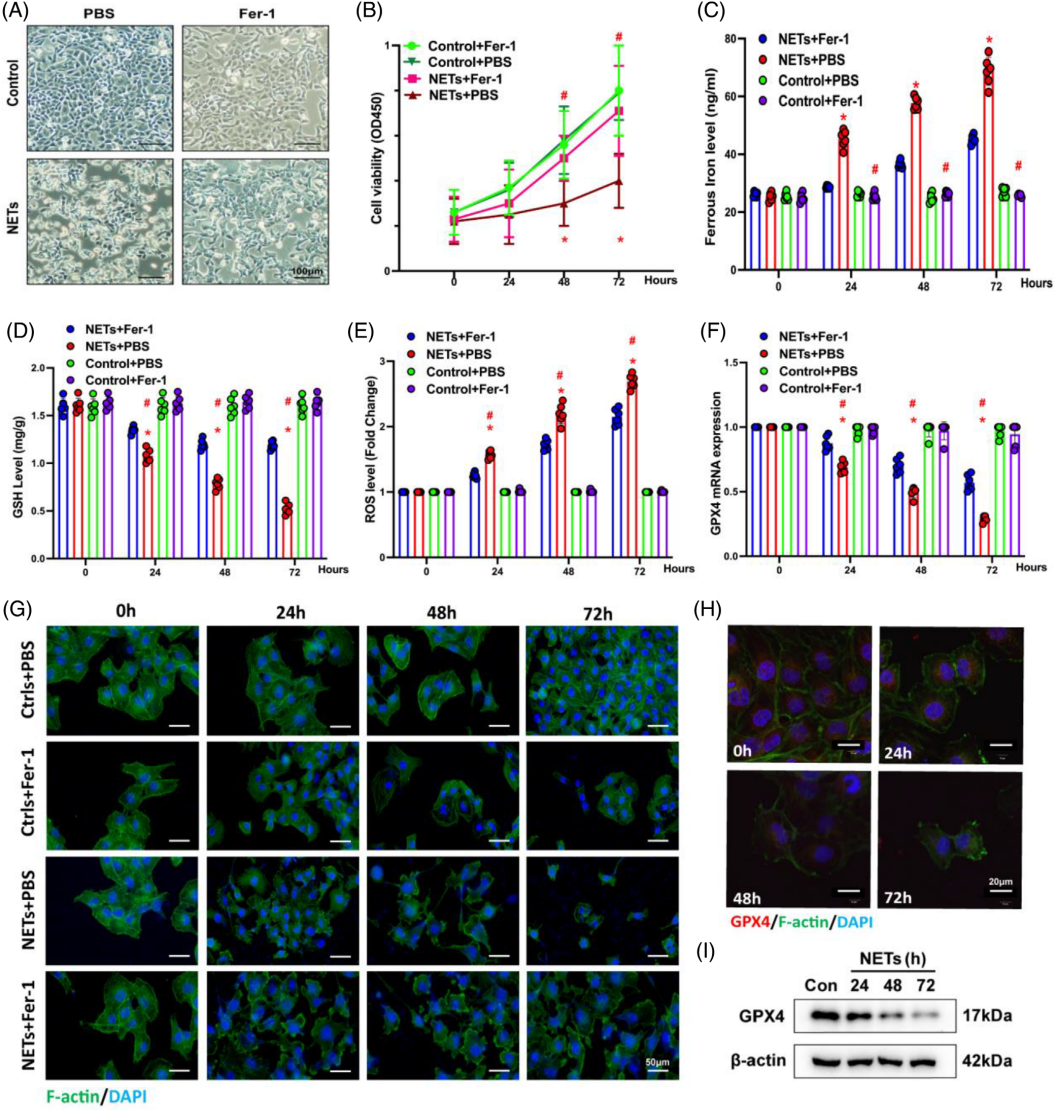
Neutrophil extracellular traps (NETs) induce ferroptosis in alveolar epithelial cells and impair cell viability. (A) Representative images of control and NET‐treated human alveolar epithelial cells (HPAEpiC) cells with or without administering ferroptosis inhibitor Fer‐1 (scale bar = 100 μm). (B) Cell viability was measured by Cell Counting Kit‐8 (CCK‐8) assay. (C) An iron assay kit measured ferrous iron (Fe^2+^) levels. (D) ELISA measured the level of glutathione (GSH). (E) The level of reactive oxygen species (ROS) was detected by DCF‐DA assay. (F) Real‐time quantitative PCR (RT‐qPCR) measured the glutathione‐peroxidase 4 (GPX4) mRNA levels. (G) Representative immunofluorescence (IF) images showed the time‐dependent morphological changes of HPAEpiC cells (scale bar = 50 μm). (H and I) The GPX4 protein and mRNA levels were analysed by IF and Western blotting, respectively. ^*^ *p* < .05. ^#^NETs + Fer‐1 versus NETs + PBS (two‐way analysis of variance [ANOVA] with Tukey's correction).

The performance of the lungs relies on alveolar epithelial cells. Unfortunately, these cells are prone to damage in the case of ALI. Next, we investigated the correlation between ferroptosis and alveolar epithelial cell damage induced by NETs in vitro. HPAEpiC cells treated with NETs for 24 h showed cell death morphological changes (Figure 3A) and decreased viability (Figure 3B). In contrast, these changes were reversed by ferroptosis inhibitor ferrostatin‐1 (Fer‐1), suggesting the involvement of ferroptosis in NET‐induced injury. Consistent with previous findings in vivo, the levels of ferrous iron (Figure 3C) and ROS (Figure 3E) increased in a time‐dependent manner, and the level of GSH (Figure 3D) and GPX4 mRNA expression (Figure 3F) decreased in NET‐treated cells, which was reversed by Fer‐1. Based on the IF results, notable morphological changes were observed over time in HPAEpiC cells treated with NETs. These changes were characterised by damage to the cell cytoskeleton, which was effectively prevented by Fer‐1 treatment (Figure 3G). In addition, NETs impacted GPX4 expression in vitro (Figure 3H). When HPAEpiC cells were treated with NETs, GPX4 expression showed a time‐dependent reduction (Figure 3I). The above results indicate that NET‐induced ferroptosis in alveolar epithelial cells contributes to SI‐ALI in vitro.

### 3.5 NETs induce ferroptosis in alveolar epithelial cells via activation of METTL3‐mediated m6A modification

**FIGURE 4:**
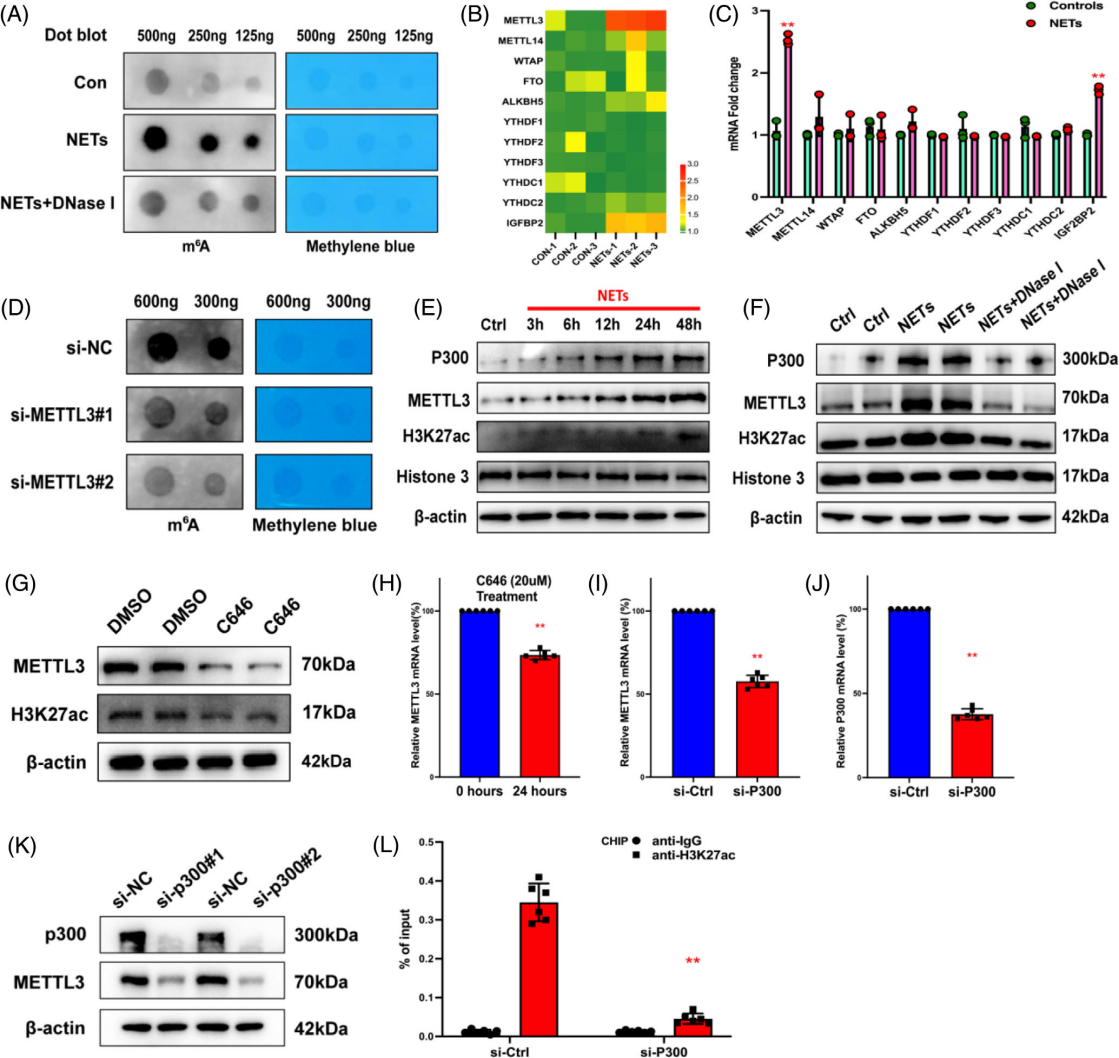
Neutrophil extracellular traps (NETs) promote METTL3 transcription via activation of H3K27ac signalling, catalysed by p300. (A and D) The level of m6A modification was analysed by dot blot assay. (B) RNA sequencing (RNA‐seq) identified the upregulation of METTL3 in NET‐treated alveolar epithelial cells. (C) Real‐time quantitative PCR (RT‐qPCR) confirmed the upregulation of METTL3 and IGF2BP2. (E and F) Western blot analysis shows that NETs increased p300, METTL3 and H3K27ac. While DNase I reversed these changes, (G) p300 inhibitor C646 reduced protein levels of H3K27ac and METTL3 in Western blot analysis. (H) C646, confirmed by RT‐qPCR, inhibited the relative METTL3 mRNA level. (I–K) Based on the Western blot and RT‐qPCR results, the protein and mRNA levels of METTL3 decreased due to the silencing of p300. (L) The chromatin immunoprecipitation (ChIP) results confirmed that the knockdown of p300 decreased the enrichment of H3K27ac signalling. ^**^ *p* < .01 (two‐way analysis of variance [ANOVA] with Tukey's correction).

To reveal the critical role of m6A methylation in NET‐induced ferroptosis, we conducted a dot blot analysis. Figure 4A shows upregulated m6A methylation levels after NET stimulation, which was reversed by DNase I (Figure 4A). Then, we conducted RNA‐seq to pinpoint the essential enzymes behind the outcomes. RNA‐seq revealed a significant increase in METTL3 and a slight rise in IGF2BP2 in cells treated with NETs (Figure 4B). The RT‐qPCR analysis showed a trend consistent with the RNA‐seq (Figure 4C). Figure 4D illustrates that when METTL3 was knocked down by transfection with siRNA, the overall m6A methylation level in HPAEpiC cells was decreased.

Data analysis from UCSC genome bioinformatics (http://UCSC Genome Browser) indicated an H3K27ac solid interaction with the METTL3 promoter, indicating that H3K27ac might regulate METTL3 transcription. As a vital acetyltransferase, p300 catalyses the production of H3K27ac. We further investigated the effects of NETs on p300 and H3K27ac levels. Western blot analysis demonstrated a time‐dependent increase in the expression of p300, METTL3 and H3K27ac (Figure 4E) after treatment with NETs, which was inhibited by DNase I (Figure 4F). C646 is an inhibitor targeting p300. Western blot analysis indicated that C646 could significantly inhibit H3K27ac and METTL3 expression (Figure 4G). Also, C646 degraded mRNA level of METTL3 after 24 h of treatment (Figure 4H). Similarly, the knockdown of p300 degraded mRNA and protein expression levels of p300 and METTL3 (Figure 4I–K). We then performed a quantitative ChIP assay to examine the enrichment of H3K27ac on the METTL3 promoter region. H3K27ac enrichment in the METTL3 promoter is downregulated when p300 is knocked down. These data confirmed that p300‐mediated H3K27ac activation in the promoter of METTL3 might partly account for the upregulation of METTL3 (Figure 4L) and play a role in NET‐mediated SI‐ALI.

**FIGURE 5:**
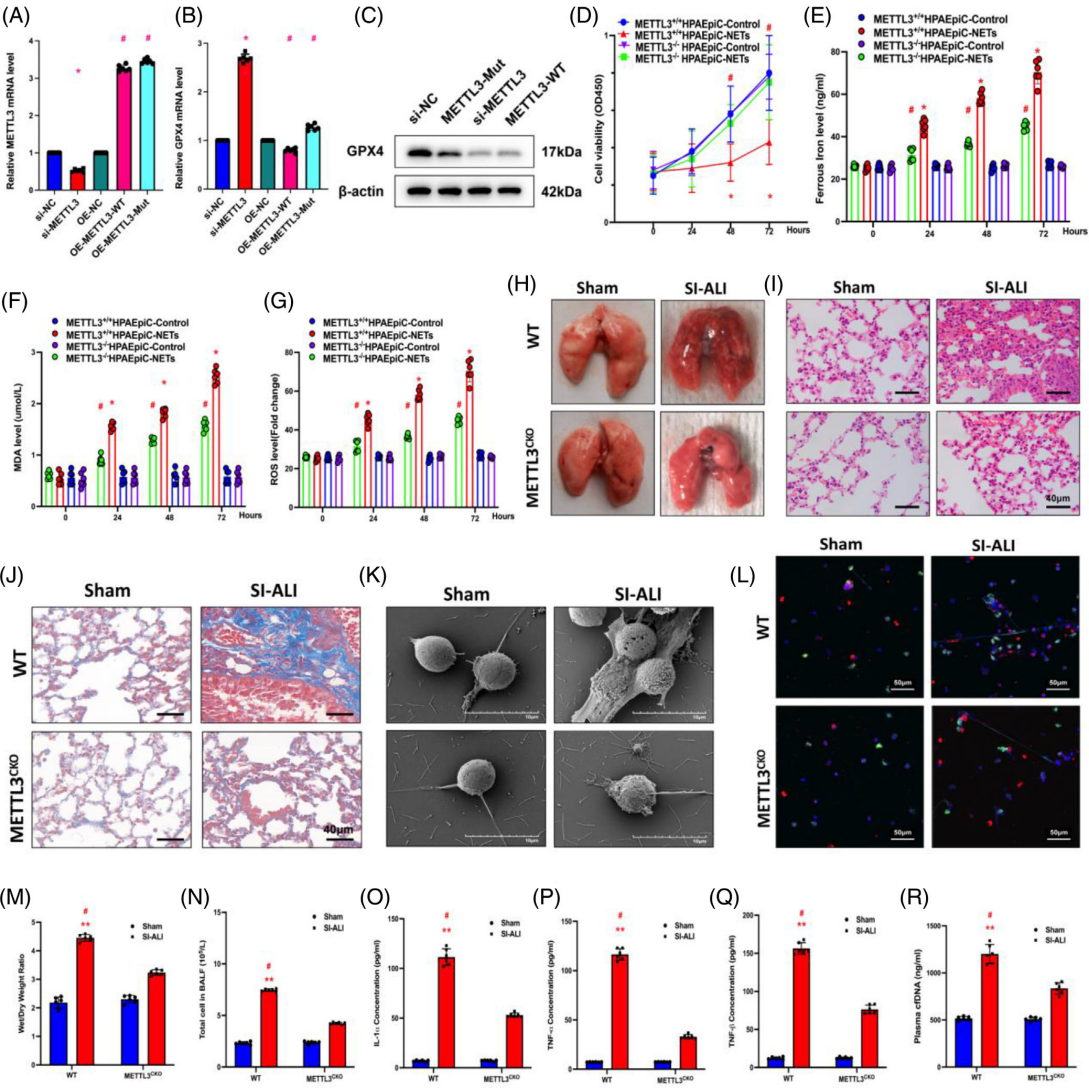
The knockout of METTL3 attenuates sepsis‐induced acute lung injury (SI‐ALI). (A and B) Real‐time quantitative PCR (RT‐qPCR) was used to evaluate METTL3 and glutathione‐peroxidase 4 (GPX4) mRNA levels in the si‐METTL3, OE‐METTL3 and OE‐METTL3‐Mut groups. (C) GPX4 protein levels in si‐NC, METTL3‐Mut, si‐METTL3 and METTL3‐WT groups. (D) Cell Counting Kit‐8 (CCK‐8) assay evaluated human alveolar epithelial cells (HPAEpiC) cell viability (*n* = 6 in each group). (E) An iron assay kit was used to measure ferrous iron (Fe^2+^) levels (*n* = 6 in each group). (F) A lipid peroxidation assay kit measured the MDA levels (*n* = 6 in each group). (G) The level of reactive oxygen species (ROS) was detected by DCF‐DA assay (*n* = 6 in each group). (H) Lung tissues from wild‐type (WT) and METTL3 CKO mice with SI‐ALI. (I) Images from haematoxylin and eosin (H&E) staining of lung tissues evaluated the degree of lung damage and inflammation. (J) Images from Masson staining of lung tissues evaluated the degree of lung fibrosis. (K) Scanning electron microscope evaluated the neutrophil extracellular trap (NET) level of NETs from WT and METTL3 CKO mice in the sham and SI‐ALI groups. (L) Immunofluorescence (IF) analysed the level of NETs from WT and METTL3 CKO mice in sham and SI‐ALI groups. (M) The degree of lung damage was evaluated by wet/dry ratio (*n* = 6 in each group). (N) Cell counting in mouse models (*n* = 6 in each group) measured total cells in bronchoalveolar lavage fluid (BALF). (O–Q) The degree of systemic inflammation was evaluated by tumour necrosis factor (TNF)‐α, TNF‐β and IL‐1α (*n* = 6 in each group). (R) The plasma cfDNA was detected in WT and METTL3 CKO mice in sham and SI‐ALI groups (*n* = 6 in each group). (N–P) ^*^ *p* < .05; ^**^ *p* < .01. ^#^SI‐ALI versus Sham (two‐way analysis of variance [ANOVA] with Tukey's correction).

To explore the involvement of METTL3‐mediated m6A modification in NET‐induced ferroptosis, we constructed siRNA‐mediated knockdown and lentivirus‐induced overexpression of METTL3 models. A METTL3 catalytic site mutation overexpressed model was also applied here. An evident decrease and increase in mRNA levels were observed when compared with those in the control group as determined by RT‐qPCR (Figure 5A). Since GPX4 is a crucial enzyme in regulating ferroptosis, we examined GPX4 levels. As predicted, silencing of METTL3 enhanced GPX4 mRNA and protein levels, while overexpression of METTL3 reversed the phenomenon (Figure 5B,C). We used CRISPR/Cas9 to generate METTL3 KO (METTL3^–/–^) HPAEpiC cells to detect ferroptosis‐associated changes. METTL3^–/–^ cells and METTL3^+/+^ cells were treated with NETs for 24 h to induce ferroptosis. Compared to METTL3^+/+^ cells, METTL3^–/–^ cells showed enhanced cell viability (Figure 5D) accompanied by lower ferrous iron (Figure 5E), MDA levels (Figure 5F) and ROS levels (Figure 5G). In summary, NETs activated p300 and further stimulated H3K27ac‐mediated METTL3 transcription, which induced ferroptosis in alveolar epithelial cells.

### 3.6 METTL3 KO inhibits NET‐induced ALI in mice

Based on the above results, we have confirmed the involvement of METTL3 in NET‐induced ferroptosis in alveolar epithelial cells. Since ferroptosis exerted significant effects during the progression of SI‐ALI, we next asked whether METTL3 conditional KO could attenuate the degree of lung damage and inflammation. The strategies for generated METTL3 CKO mice are shown in Figure S4A. The expression levels of METTL3 in lung are validated by Western blot (Figure S4B) and lung tissue Immunohistochemistry (IHC) (Figure S4C). As expected, METTL3 CKO mice showed lower lung injury degrees than WT (METTL3*^flox/flox^*) mice after CLP (Figure 5H). This was evidenced by decreased inflammatory cell infiltration (Figure 5I), mild pulmonary fibrosis (Figure 5J) and a lower wet/dry weight ratio (Figure 5M). Furthermore, the number of cells and inflammatory cytokines in BALF samples (TNF‐α, TNF‐β, IL‐1α) were decreased in METTL3 CKO mice, suggesting a reduction in lung inflammation (Figure 5N–Q). Additionally, scanning electron microscopy (SEM) (Figure 5K) and IF assays (Figure 5L) demonstrated a decreased release of NETs from neutrophils in METTL3 CKO mice. These findings were further confirmed by lower plasma cfDNA concentrations in METTL3 CKO mice as determined by ELISA (Figure 5R). Also, the survival rates of the METTL3 CKO group were higher than those of WT mice after CLP (Figure S5A). Collectively, METTL3 KO attenuated NET‐induced ALI in mice.

### 3.7 METTL3 induces HIF‐1α upregulation via m6A‐IGF2BP2‐dependent mechanism to exacerbate ferroptosis

**FIGURE 6:**
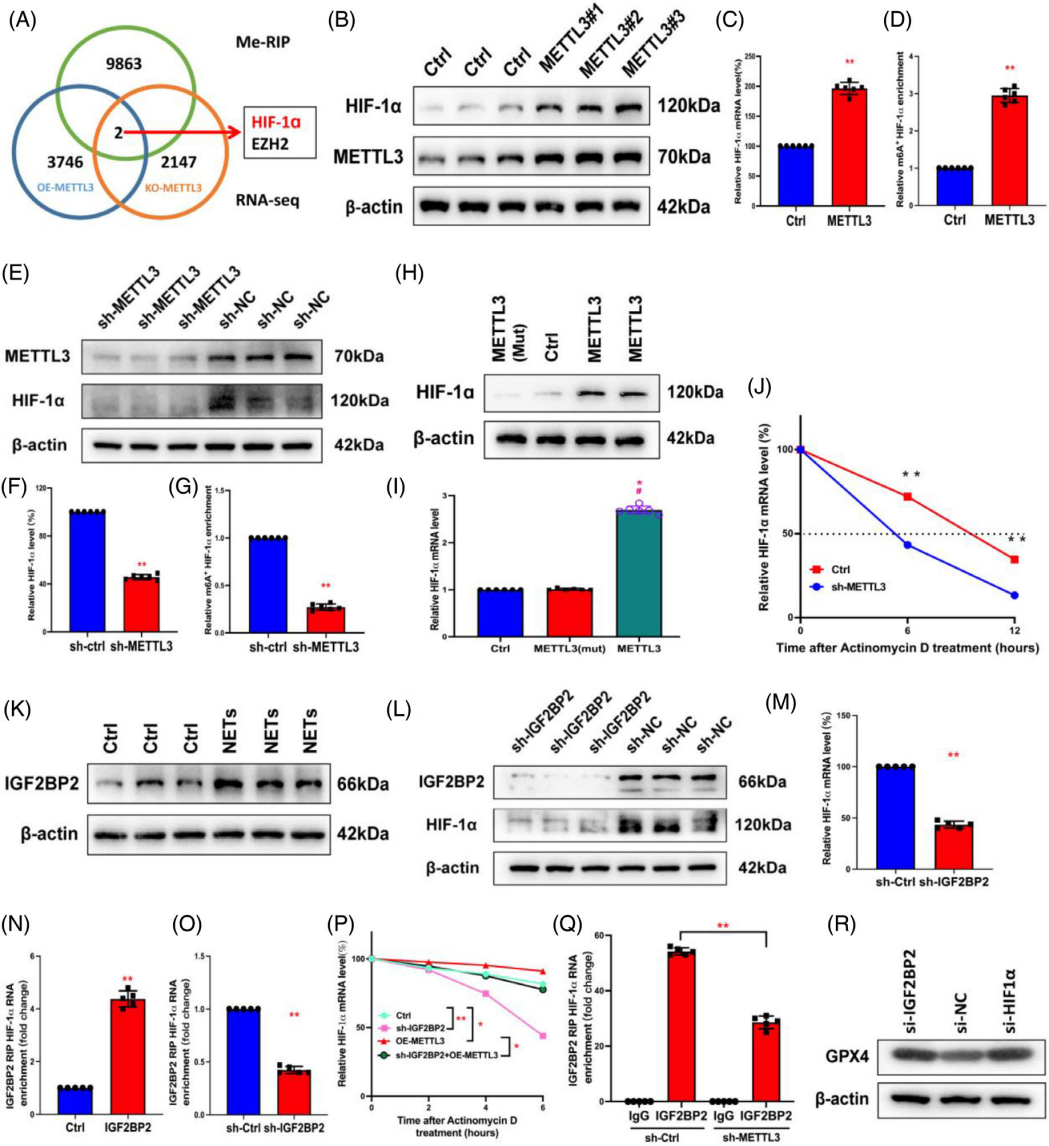
METTL3 mediates m6A modification of hypoxia‐inducible factor‐1α (HIF‐1α) depending on IGF2BP2. (A) RNA sequencing (RNA‐seq) and MeRIP‐seq were used to identify differentially expressed genes in METTL3 overexpression and knockout cells compared to their corresponding controls. (B) In control and overexpressing‐METTL3 groups, Western blot evaluated the METTL3 and HIF‐1α. (C) Relative HIF‐1α mRNA level was evaluated by real‐time quantitative PCR (RT‐qPCR) in overexpressing‐METTL3 and control groups (*n* = 6 in each group). (D) MeRIP‐qPCR analysis evaluated the m6A modification of HIF‐1α level in controls and METTL3‐overexpressing groups (*n* = 6 in each group). (E–G) In sh‐METTL3 and sh‐NC groups, Western blot evaluated the METTL3 and HIF‐1α. RT‐qPCR evaluated the relative HIF‐1α mRNA level. MeRIP‐qPCR analysis was used to evaluate the m6A modification of the HIF‐1α level (*n* = 6 in each group). (H and I) The mRNA and protein levels of HIF‐1α were evaluated by RT‐qPCR and Western blot, respectively, in METTL3‐Mut, controls and METTL3 group (*n* = 6 in each group). (J) Relative HIF‐1α RT‐qPCR evaluated mRNA level in METTL3 knockout group and wild type (WT) (*n* = 6 in each group). (K) Western blot analysis revealed an increase in IGF2BP2 after neutrophil extracellular trap (NET) stimulation. (L and M) Western blot and RT‐qPCR evaluated HIF‐1α protein and mRNA levels in the sh‐IGF2BP2 group and controls. The dashed line shows the half‐life of HIF‐1α in response to actinomycin D. (N and O) RIP assays showed an upregulated and downregulated interaction between HIF‐1α and IGF2BP when IGF2BP2 was overexpressed and silenced. (P) RT‐qPCR evaluated relative HIF‐1α mRNA level in control, sh‐IGF2BP2, OE‐METTL3 and sh‐IGF2BP2 + OE‐METTL3 groups after actinomycin D treatment (*n* = 6 in each group). (Q) RIP assays showed the interaction between HIF‐1α and IGF2BP when METTL3 was silenced. (R) The protein level of glutathione‐peroxidase 4 (GPX4) was increased in the sh‐IGF2BP2 group and sh‐HIF‐1α group via Western blot. ^**^ *p* < .01 (two‐way analysis of variance [ANOVA] with Tukey's correction).

To identify the molecular mechanisms related to METTL3‐regulated ferroptosis, we conducted RNA‐seq in HPAEpiC cells with METTL3 overexpression and KO levels (Specimen 5 vs.5). RNA‐seq results showed that 3748 transcripts were significantly upregulated in the METTL3 overexpression group and 2149 transcripts were significantly downregulated in the METTL3 knockdown group compared with the corresponding control in each group. We also performed MeRIP‐seq in HPAEpiC cells with METTL3 overexpression and control cells. The analysis revealed that m6A peaks of 9865 transcripts showed increased abundance. It is worth noting that the RNA‐seq and MeRIP‐seq data had two transcripts that overlapped, originating from two distinct genes, one of which was HIF‐1α (Figure 6A). Then, we validated the protein and mRNA levels of HIF‐1α in METTL3‐overexpressing cells (Figure 6B,C). Overexpression of METTL3 significantly upregulated the expression of HIF‐1α. In lentivirus‐transfected METTL3 knockdown cells, downregulated METTL3 significantly reduced HIF‐1α mRNA and protein levels (Figure 6E,F). With the overexpression and KO of METTL3, the proportion of HIF‐1α mRNA with m6A methylation increased (Figure 6D) and decreased accordingly (Figure 6G). However, no upregulation existed in cells with catalytic mutant METTL3 (Figure 6H,I). Meanwhile, we constructed mutant plasmids to detect the predicted m6A site to determine the specific modification of HIF‐1α (Figure S6A,B). We used actinomycin D to investigate the impact of METTL3‐mediated m6A modification on HIF‐1α mRNA stability. The results showed that overexpression of METTL3 significantly inhibited the degradation of HIF‐1α mRNA (Figure 6J).

Previous results demonstrated that NETs could increase the level of IGF2BP2, an m6A‐binding protein that increases mRNA's stability and translation.^35^ Thus, we speculated that NETs could stimulate HIF‐1α via an m6A‐IGF2BP2‐dependent mechanism. As we expected, stimulation of NETs upregulated the expression of IGF2BP2 in vitro (Figure 6K). In addition, silencing of IGF2BP2 significantly reduced the protein and mRNA level of HIF‐1α (Figure 6L,M). We further evaluated the interaction between HIF‐1α and IGF2BP2 by RIP. Overexpression and knockdown of IGF2BP2, respectively, upregulated and downregulated the HIF‐1α mRNA interacted with IGF2BP2 (Figure 6N,O). The actinomycin D assay showed that overexpression of METTL3 could partially restore the decreased stability of HIF‐1α caused by knockdown of IGF2BP2 (Figure 6P). Meanwhile, the results of RIP indicated that knockdown of METTL3 reduced the interaction between IGF2BP2 and HIF‐1α mRNA. As shown in Figure S6C, m6A mutant sites in the HIF‐1α transcripts limited the binding of IGF2BP2. Moreover, METTL3 knockdown inhibited the binding interaction between IGF2BP2 and HIF‐1α mRNA (Figure 6Q), which suggests that IGF2BP2 could bind HIF‐1α mRNA in a METTL3/m6A‐dependent manner. Moreover, the knockdown of IGF2BP2 and HIF‐1α promoted GPX4 expression (Figure 6R), suggesting that METTL3 enhanced HIF‐1α expression via an m6A‐IGF2BP2‐dependent mechanism, which further enabled ferroptosis via GPX4 downregulation.

### 3.8 METTL3 induces enhanced glycolysis and decreased oxidative phosphorylation in alveolar epithelial cells

**FIGURE 7:**
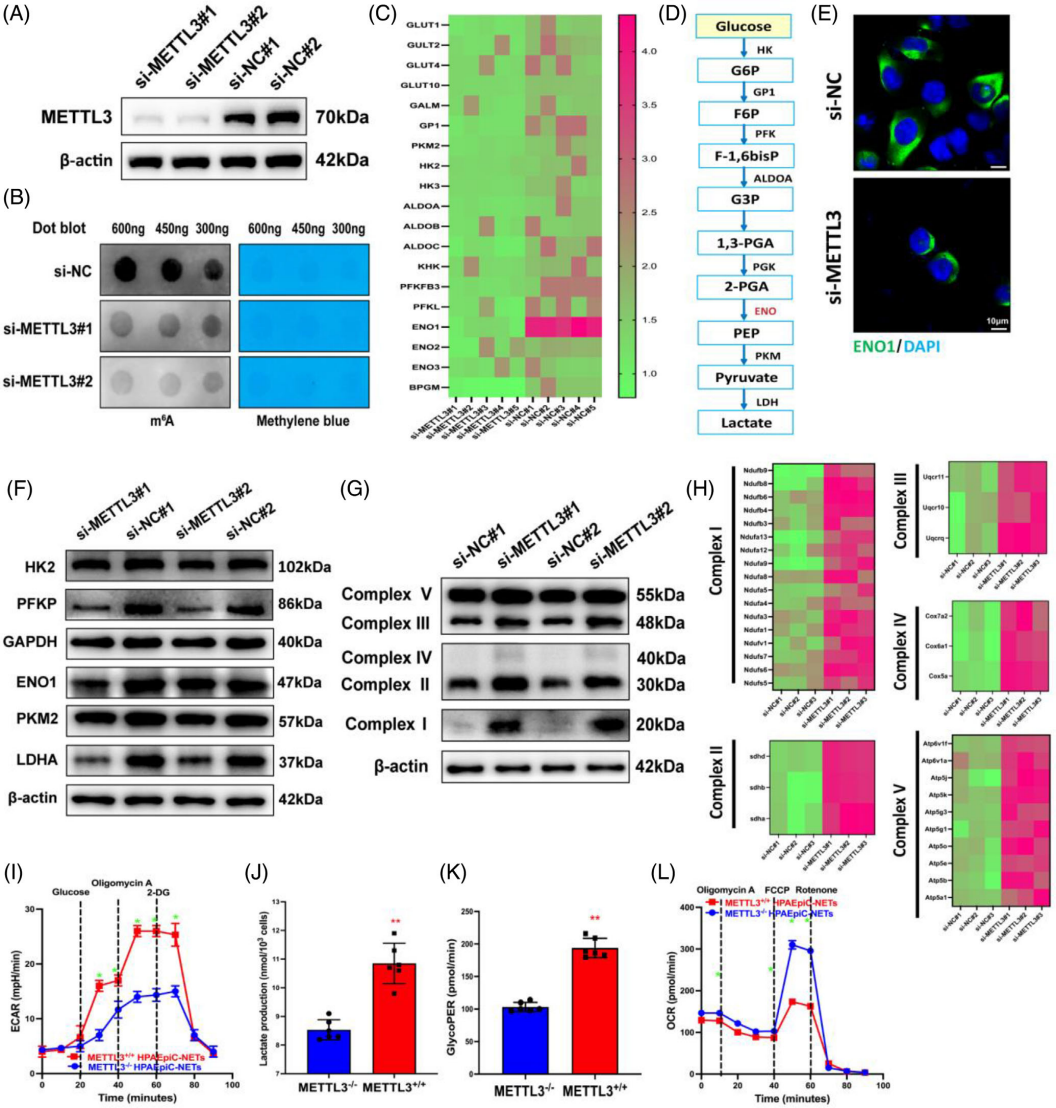
METTL3 induces enhanced glycolysis and decreased oxidative phosphorylation. (A) Western blot analysis verifies the efficiency of knockdown METTL3. (B) The level of m6A modification was analysed by dot blot assay. (C) RNA sequencing (RNA‐seq) evaluated glycolysis‐associated genes in METTL3 knockdown groups and controls. (D) A simplified metabolic flow diagram of glycolysis. (E) The level of enolase 1 (ENO1) was evaluated by immunofluorescence (IF) assay in METTL3 knockdown groups and controls. (F) The protein levels of glycolytic enzymes were evaluated by Western blot in METTL3 knockdown groups and controls. (G) Complex I∼V‐associated protein expression levels were evaluated by Western blotting in METTL3 knockdown groups and controls. (H) RNA‐seq evaluated complex I∼V‐associated genes in mitochondria in METTL3 knockdown groups and controls. (I) Extracellular acidification rate (ECAR) evaluated the degree of glycolysis in METTL3^+/+^ and METTL3^−/−^ cells treated with neutrophil extracellular traps (NETs) over time. (J and K) Lactate and glycoPER production evaluated the degree of glycolysis in METTL3^+/+^ and METTL3^−/−^ cells treated with NETs. (L) The oxygen consumption rate (OCR) evaluated the degree of aerobic glucose metabolism in METTL3^+/+^ and METTL3^−/−^ cells treated with NETs over time. ^**^ *p* < .01 (two‐way analysis of variance [ANOVA] with Tukey's correction).

HIF‐1α is a master controller of cell glycolysis, and excessive glycolysis may play a critical role in the progression of ALI.^17^, ^36^ We hypothesised that METTL3 may affect ferroptosis in alveolar epithelial cells by regulating metabolic pathways. An RNA‐seq analysis was conducted between cells pretreated with si‐METTL3 and si‐NC. Western blotting and dot blotting verified the knockdown levels of METTL3 and the corresponding downregulation of m6A levels, respectively (Figure 7A,B). We focused on the molecules related to metabolism in RNA‐seq (Figure 7C). Results of the differential analysis showed that ENO1, a glycolytic enzyme that catalyses the conversion of 2‐poly‐γ‐glutamic acid into phosphoenolpyruvate (Figure 7D), was significantly downregulated in si‐METTL3 groups compared with control groups (Figure 7C), which was validated by Western blotting and IF (Figure 7E,F). Moreover, the silencing of METTL3 decreased the expression levels of several glycolytic enzymes, such as HK2, PFKP, PKM2 and LDHA (Figure 7F). Then, we asked whether aerobic oxidation, an indicator of glucose metabolism, changed during METTL3 silencing. We measured complex I∼V protein levels in METTL3 knockdown or normal cells for this (Figure 7G). Knockdown of METTL3 significantly elevated the expression of mitochondrial complex, which is consistent with the RNA‐seq results that complex I∼V‐associated mRNA expression in the mitochondria was noticeably higher in si‐METTL3 cells than in controls (Figure 7H), suggesting that METTL3 could inhibit oxidative phosphorylation. These results were further verified by Western blot analysis. The levels of glycolysis‐associated markers, such as ECAR (Figure 7I), lactate production (Figure 7J) and glycoPER (Figure 7K), were all significantly enhanced in NET‐treated METTL3^+/+^ HPAEpiC cells. In contrast, indicators of oxidative phosphorylation, such as OCR (Figure 7L), basal respiration, maximum respiration and spare respiratory capacity (Figure S6), were all inhibited in METTL3^+/+^ HPAEpiC cells. Herein, we confirm that METTL3 induces enhanced glycolysis and decreased oxidative phosphorylation in alveolar epithelial cells.

### 3.9 Inhibiting NET formation via PAD4 KO attenuates ferroptosis and sepsis‐associated lung damage in mice

**FIGURE 8:**
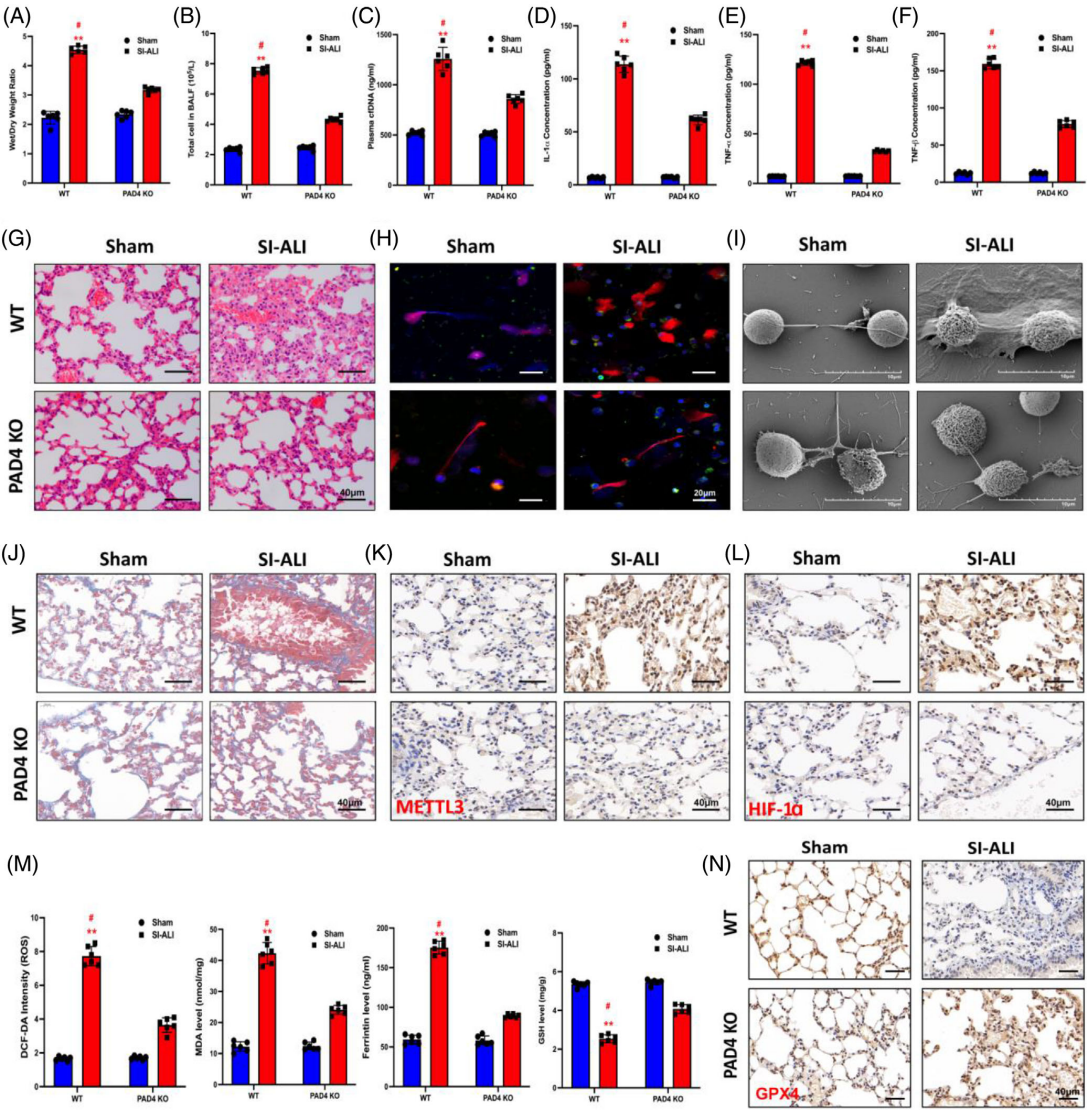
Peptidyl arginine deiminase 4 (PAD4) knockout alleviates ferroptosis in alveolar epithelial cells and sepsis‐induced acute lung injury (SI‐ALI). (A) The degree of lung damage was evaluated by wet/dry ratio from wild‐type (WT) and PAD4 knockout (KO) mice in sham and SI‐ALI groups (*n* = 6 in each group). (B) Cell counting (*n* = 6 in each group) measured total cells in bronchoalveolar lavage fluid (BALF). (C) Plasma cfDNA evaluated neutrophil extracellular trap (NET) formation from WT and PAD4 KO mice in sham and SI‐ALI groups (*n* = 6 in each group). (D–F) Interleukin (IL)‐1α, tumour necrosis factor (TNF)‐α and TNF‐β evaluated the degree of systemic inflammation. (G) Images from haematoxylin and eosin (H&E) staining of lung tissues evaluated the degree of lung damage. Images from immunofluorescence (IF) assay (H) and scanning electron microscopy (SEM) (I) evaluated the level of NETs. (J) The degree of lung fibrosis was evaluated by Masson staining analysis. The levels of METTL3 (K), hypoxia‐inducible factor‐1α (HIF‐1α) (L) and glutathione‐peroxidase 4 (GPX4) (N) were evaluated by IHC. (M) The level of ferroptosis was evaluated by reactive oxygen species (ROS), MDA, ferritin and glutathione (GSH) (*n* = 6 in each group). ^**^ *p* < .01. ^#^SI‐ALI versus Sham (two‐way analysis of variance [ANOVA] with Tukey's correction).

Since we demonstrated that inhibition of NETs through weakening PAD4 activity could mitigate ferroptosis and attenuate SI‐ALI, we next investigated the role of PAD4 KO in ALI progression using a CLP‐induced SI‐ALI model. PAD4 KO inhibited NET formation according to IF assays (Figure 8H) and SEM images (Figure 8I), and lower levels of cfDNA were also detected in PAD4 KO mice than in WT mice (Figure 8C). Consistent with our previous observations, PAD4 KO decreased lung damage and inflammation. This was evidenced by a lower wet/dry weight ratio (Figure 8A), reduced levels of total cells in BALF and reduced plasma cfDNA (Figure 8B,C) and less inflammatory cell infiltration in lung tissues (Figure 8G). Moreover, systemic inflammation was alleviated by inhibiting NETs, as lower concentrations of IL‐1α, TNF‐α and TNF‐β were observed in PAD4 KO mice than in WT mice (Figure 8D–F). Also, PAD4 KO mice lung tissues showed mild NET formation and mild fibrosis compared to those of WT mice (Figure 8H–J). As a result of decreased expression levels of METTL3 and HIF‐1α in lung tissues from PAD4 KO mice (Figure 8K,L), we next measured ferroptosis‐ and oxidation‐associated markers and found that the levels of ROS, MDA and ferritin were reduced and that the levels of GSH and GPX4 were enhanced after PAD4 KO (Figure 8M,N). In conclusion, inhibition of NET formation via PAD4 depletion could attenuate ferroptosis and SI‐ALI.

## 4 DISCUSSION

In the present study, we confirmed that NET‐activated METTL3 exacerbated ferroptosis via m6A‐IGF2BP2‐dependent mitochondrial metabolic reprogramming during the progression of sepsis‐associated lung injury. First, enhanced levels of NETs and ferroptosis were detected in SI‐ALI mice, which were reversed by inhibitors of NETs, such as Cl‐amidine, anti‐Ly6G or DNase I. Meanwhile, the degree of lung damage and inflammatory response were also alleviated after these treatments. Moreover, Fer‐1, the ferroptosis inhibitor, also attenuated alveolar epithelial damage induced by NETs. Next, we performed RNA‐seq and RT‐qPCR, which, combined with data from the UCSC genome bioinformatics site, demonstrated that NETs induced p300‐activated H3K27ac signalling to promote METTL3 transcription. Higher cell viability and decreased level of ferroptosis were found in METTL3^−/−^ cells treated with NETs than in METTL3^+/+^ cells. And we also demonstrated that METTL3‐conditioned KO decreased NET formation and alleviated lung damage in vivo. Finally, we used RNA‐seq and MeRIP‐seq to determine that METTL3‐induced HIF‐1α expression depends on the binding of m6A and IGF2BP2. Since HIF‐1α was known as a central regulator of glycolysis to mitigate the effects of hypoxia,^37^ we next evaluated metabolism‐associated genes by RNA‐seq. Upregulated glycolysis and downregulated oxidative phosphorylation were found in METTL3^+/+^ cells compared with METTL3^−/−^ ones. These results indicated that NET‐induced METTL3 upregulation in alveolar epithelial cells exacerbated ferroptosis via m6A‐IGF2BP2‐dependent mitochondrial metabolic reprogramming.

SI‐ALI is a lethal illness worldwide.^38^ During its progression, neutrophils can release NETs, web‐like structures comprised of DNA, histones, MPO, cathepsin G and other antibacterial substances, to neutralise and kill pathogens.^39^ However, NET components can also serve as DAMPs, which may induce a persistent inflammatory response and tissue damage.^40^ According to our previous study, NETs aggravated systemic inflammation via the release of TNF‐1α, IL‐1β and IL‐6,^26^ and such inflammatory cytokines could further induce NET formation, forming a vicious circle.^41^ NETs can also interact with platelets, triggering extensive ‘immunothrombosis’.^42^ Our previous study demonstrated that neutrophils released tissue factor‐enriched NETs, which correlated with enhanced thrombosis and worse clinical outcomes.^43^

M6A is the most prevalent and conserved type of internal mRNA modification. Emerging evidence has demonstrated its involvement in various biological processes.^44^ Many studies have indicated that m6A participated in cancer pathogenesis and progression by regulating tumour‐associated gene expression.^45^ Additionally, accumulating evidence has uncovered a crucial role of m6A during sepsis.^24^, ^26^ Our previous work confirmed that NETs induced METTL3‐mediated m6A modification of GPX4 via TLR9/MyD88/NF‐κB pathway in sepsis‐associated lung injury.^16^ In our present study, we explored a new mechanism by which NETs could activate METTL3 transcription through p300‐mediated H3K27ac upregulation. And our further study revealed the exact pathway by which alveolar epithelial cell undergoes ferroptosis via NET‐mediated m6A modification.

Notably, NET‐induced metabolic reprogramming has gradually drawn researchers’ attention in recent years. Aldabbous et al.^46^ demonstrated that NETs induced a pro‐inflammatory and pro‐angiogenesis response, characterised by enhanced glycolysis, in human pulmonary artery endothelial cells.^47^ Moreover, enhanced glycolysis induced a pro‐inflammatory phenotype in endothelial and alveolar epithelial cells during sepsis.^17^, ^36^ We also overviewed the mutual promotion between glycolysis and inflammation/ROS production.^41^ Intriguingly, ferroptosis, as a newly emerged form of necrosis, results from ROS accumulation and the subsequent iron‐dependent lipid peroxidation,^38^, ^48^ which suggests the involvement of metabolic reprogramming.

During sepsis, an imbalance between ROS production and clearance is one of the main mechanisms of ferroptosis.^37^, ^38^ Increasing evidence suggests the critical role of metabolic reprogramming in regulating ferroptosis and innate inflammatory response, but the underlying mechanism remains unclear. According to our speculation, we focused on the metabolic reprogramming induced by METTL3‐mediated m6A modification. Decreased levels of glycolytic enzyme genes and increased levels of complex I∼V‐associated genes in mitochondria in METTL3 knockdown cells indicated that m6A modification of HIF‐1α caused increased glycolysis and decreased oxidative phosphorylation. These metabolic changes might further contribute to ROS accumulation and ferroptosis in alveolar epithelial cells. Finally, PAD4 KO was confirmed to decrease ferroptosis and reduce lung damage. These findings shed light on a novel mechanism for metabolic control of inflammation by regulating NET release and highlight the importance of targeting aerobic glycolysis in treating sepsis and other inflammatory diseases.

There are many strengths in our study. First, we conducted a translational exploration of the role of NETs in SI‐ALI. In addition, many gene KO or overexpression mice and cells were used to test our hypothesis. Second, RNA‐seq and MeRIP‐seq were used to confirm the downstream genes of METTL3 and the expression levels of metabolism‐associated genes. Undeniably, there are still some limitations in our study. First, the underlying molecular mechanisms of the inflammatory response, ROS accumulation, and the following ferroptosis induced by enhanced glycolysis still need to be elucidated. Second, the anti‐Ly6G we used is not specific to neutrophils since it can also cause eosinophil depletion. We solved this issue via the use of pure neutrophils. In this current study, we did not specify the exact protein that undergoes regulation in the mitochondrial complex, which requires further investigation.

In conclusion, NETs lead to a rise in p300, which catalyses H3K27ac‐mediated METTL3 transcription and further contributes to m6A‐IGF2BP2‐dependent mitochondrial reprogramming that enhances ferroptosis during SI‐ALI. Targeting NETs/METTL3/ferroptosis can alleviate lung damage and systemic inflammation, suggesting their potential clinical value.

## CONFLICT OF INTEREST STATEMENT

The authors declare they have no conflicts of interest.

## Supporting information

Supporting Information

## Data Availability

All the data are requested and available to Professor Changhong Miao.
