# Supplementary material for: METTL3‐mediated N6‐methyladenosine exacerbates ferroptosis via m6A‐IGF2BP2‐dependent mitochondrial metabolic reprogramming in sepsis‐induced acute lung injury
Source: Clin Transl Med. 2023 Sep 15;13(9):e1389. doi: 10.1002/ctm2.1389 (PMC10504453; doi:10.1002/ctm2.1389)
Supplement: Supplementary file 1 — Supporting Information [file CTM2-13-e1389-s001.docx]

Supplementary Table 1. List of kits used for testing.

| Indicators | Kits | Brands |
| --- | --- | --- |
| ds-DNA | Quant-iT™ PicoGreen Kit | Invitrogen |
| MPO-DNA complexes | MPO ELISA Kit | ab119605, Abcam |
| TNF-α | TNF-α ELISA Kit | abs520010, Absin |
| IL-1β | IL-1β ELISA Kit | abs520001, Absin |
| IL-6 | IL-6 High Sensitivity ELISA Kit | abs552805, Absin |
| Ferrous iron | Iron Assay Kit | ab83366, Abcam |
| Ferritin | Ferritin ELISA Kit | ab157713, Abcam |
| MDA | Lipid Peroxidation (MDA) Assay Kit | ab118970, Abcam |
| GSH | Glutathione Assay Kit | ab65322, Abcam |
| ROS | ROS Assay Kit | S0033S, Beyotime |

Supplementary Table 2. The primer sequence of si-RNA.

|  | Sense | Antisense |
| --- | --- | --- |
| si-P300#1 | CGACUUACCAGAUGAAUUAAU | UAAUUCAUCUGGUAAGUCGUG |
| si-P300#2 | GAUGAAUUAAUCAACUCUACA | UAGAGUUGAUUAAUUCAUCUG |
| si-METTL3#1 | GCAGUUCCUGAAUUAGCUACA | UAGCUAAUUCAGGAACUGCUG |
| si-METTL3#2 | GAGGCAGCAUUGUCUCCAACC | UUGGAGACAAUGCUGCCUCUG |
| si-IGF2BP2 | GCAUGAUUCUUGAAAUCAUGC | AUGAUUUCAAGAAUCAUGCGG |
| si-HIF-1α | CGAUGGAAGCACUAGACAAAG | UUGUCUAGUGCUUCCAUCGG |

Supplementary Table 3. Primer sequence of RT-qPCR.

| Gene | Forward | Reverse |
| --- | --- | --- |
| GPX4 | GAGGCAAGACCGAAGTAAACTAC | CCGAACTGGTTACACGGGAA |
| METTL3 | CATTGCCCACTGATGCTGTG | AGGCTTTCTACCCCATCTTGA |
| METTL14 | GAACACAGAGCTTAAATCCCCA | TGTCAGCTAAACCTACATCCCTG |
| WTAP | ACTGGCCTAAGAGAGTCTGAAG | GTTGCTAGTCGCATTACAAGGA |
| FTO | ACTTGGCTCCCTTATCTGACC | TGTGCAGTGTGAGAAAGGCTT |
| ALKBH5 | CGGCGAAGGCTACACTTACG | CCACCAGCTTTTGGATCACCA |
| YTHDF1 | ACCTGTCCAGCTATTACCCG | TGGTGAGGTATGGAATCGGAG |
| YTHDF2 | CCTTAGGTGGAGCCATGATTG | TCTGTGCTACCCAACTTCAGT |
| YTHDF3 | GGTGTATTTAGTCAACCTGGGG | AAGAGAACTAGGTGGATAGCCAT |
| YTHDC1 | GAGGGCCAAATCTCCTACGC | GTCTCATGGTCAGAGCCATATTC |
| YTHDC2 | AGGACATTCGCATTGATGAGG | CTCTGGTCCCCGTATCGGA |
| IGF2BP2 | AGCTAAGCGGGCATCAGTTTG | CCGCAGCGGGAAATCAATCT |
| HIF-1α | GAACGTCGAAAAGAAAAGTCTCG | CCTTATCAAGATGCGAACTCACA |
| β-actin | CATGTACGTTGCTATCCAGGC | CTCCTTAATGTCACGCACGAT |

Supplementary Table 4. CHIP primer

| METTL3 | Forward | TCCAGCTTCTTTCCAATGCC |
| --- | --- | --- |
| METTL3 | Reverse | TTTGACTGGCATGGCTCCTG |
